# Can optic disc vessel density help in cases of residual disc elevation after shunt surgery in cases of idiopathic intracranial hypertension?

**DOI:** 10.1007/s10103-024-04064-5

**Published:** 2024-05-07

**Authors:** Nermien Salah El-Dien Mohammed El-Haddad, Shymaa Adel Ismael, Nehal Shabaan, Yasser Ghoraba, Eman A. Elhamrawy, Nashwaa Lamie, Fatma Atwaa, Sanna Ahmed Mohamed, Mona Nabeh Mansour

**Affiliations:** 1 Faculty of Medicine, Al-Azhar University, Cairo, Egypt; 2 Faculty of medicine, Tanta university, Tanta, Egypt; 3 Al-Zahraa Hospital, Cairo, Egypt

**Keywords:** Papilledema, Idiopathic intracranial hypertension, Optic disc vessel density

## Abstract

**Aim:**

To detect if we can use the reduction in the optic disc vessel density as an indicator to the reduction in intracranial tension in patients with residual optic disc elevation after shunt surgery as fundus examination in those cases is not conclusive.

**Patients and method:**

21 patients with papilledema due to idiopathic intracranial hypertension underwent shunt surgery. Full neurological and ophthalmological assessments were done. The optic disc vessel density was measured before and 3 months after surgery. Patients were then divided according to the resolution of papilledema into 2 groups: 1) Residual disc elevation group. 2) Completely resolved disc edema group. CSF pressure was measured via lumber puncture preoperative for all patients and 3 months post-operative only for patients with residual disc edema. A comparison between both groups was done.

**Results:**

There was a highly statistically significant difference between the two groups as regard the papilledema grade (the residual disc elevation group had a higher grade of papilledema) with P-value=0.000. As regard the difference in the preoperative optic disc vessel density between the two groups, there were statistically significant differences (optic disc vessel density was more in the residual disc elevation group). As regard the postoperative optic disc vessel density, there were non-significant differences between the two groups in whole image, inside disc and peripapillary vessel density (either in macro or microvasculature).

**Conclusion:**

The optic disc vessel density decreased with normal postoperative CSF opening pressure in cases with residual disc elevation postoperatively. Thus, in cases of residual optic disc swelling after shunt surgery, we can detect the reduction of intracranial pressure by the reduction in the optic disc vessel density which is a safe non-invasive technique. That may help in cases of residual disc elevation.

## Introduction

Idiopathic intracranial hypertension (IIH) is a disease of increasing intracranial pressure with normal neuroimaging and cerebrospinal fluid (CSF) analysis with unknown etiology. Neurological surgeons and ophthalmologists share in the diagnosis and follow-up of management of IIH [1].

The etiology of the disease has not been fully understood yet. Diagnosis is mainly by exclusion and in accordance with Dandy criteria.

The incidence in the Middle East area is estimated to be 2.02-2.2/100,000 in general population which is higher than the Western area (0.9/100,000) [2]. Obese women have 20 times higher incidence than the normal population. The incidence in obese women is 19.4/100.000, while in the general population is 0.9–1.0/100.000 [3].

The least incidence of visual impartment after medical or surgical treatment of IIH was 13.1% of the patients and 2% of them had blindness [2, 4].

Studies observed that obesity increases the incidence of severe visual impairment. So, the obese patients should be managed strictly with close follow-up. Unfortunately, the global incidence of obesity increases that increases the incidence of IIH [2].

The features of IIH-related headache vary substantially and in the context of a limited amount of clinical studies that aim at characterizing them, the IHS-criteria remain relatively unspecific in their description. Patients commonly describe their headache as pressing, explosive with a frontal, retroorbital localization. Frequently the headache has a migraine phenotype and overuse of analgesic is observed in over a third of IIH patients .Phenotypic similarities may hamper its distinction from migraine and other headaches [5].

As the disease affects the women in the childbearing period and causes visual impairment, it is crucial to treat and follow-up patients thoroughly.

Optical coherence tomography angiography (OCTA) is a non-invasive technique that allows us to detect the vasculature of the optic disc and retinal layers. Moreover, it can measure the vessel density of the optic disc and retinal layers [6].

After shunt surgery, some patients may have residual optic disc elevation that may take several months to resolve completely or may not resolve forever. Depending on headaches only in detecting patients’ improvement is inaccurate. Measuring the opening CSF pressure is an accurate method to detect the surgery success in these cases but it is an invasive procedure. So, we try to use optic disc vessel density measurement to help in cases of residual disc elevation as fundus examination is not conclusive in those cases.

## Patients and methods

This study included 21 patients with papilledema due to idiopathic intracranial hypertension who underwent shunt surgery from December 2021 to September 2022. Patients were diagnosed and operated in a tertiary university hospital. Written informed consents were taken from all patients. The study was done in accordance to Declaration of Helsinki.

All patients were diagnosed as IIH according to the modified Dandy’s criteria: (1) signs and symptoms of increased intracranial pressure; (2) no other neurological abnormalities or impaired level of consciousness (with the exception of CN VI palsy); (3) elevated intracranial pressure (ICP) w/normal CSF composition; (4) a computed tomography (CT) scan which shows no etiology for increased ICP (the original (5) no other cause for intracranial hypertension found. Surgery was indicated in all patients.

Patient with IIH not indicated for surgery was excluded from the study. Other exclusion criteria included: any systemic disease affecting the eye , myopia of more than -6 D, medications that affect ocular blood vessels, history of ocular trauma or surgery, alcohol intake, smoking, and pregnancy because all of the above affect the optic disc vessel density.

All patients underwent history taking full neurological and ophthalmological examinations.

MRI brain, MR venography, lumber puncture for measurement of CSF pressure in left lateral decubitus position, and CSF analysis were done for all patients.

Ophthalmic examination was done in the form of: visual acuity measurements, anterior segments examination, fundus examination by 90D lens, intraocular pressure measurements, visual field examination by Humphrey Field Analyzer; (24-2 SITA; Carl-Zeiss Meditec, Dublin, CA), and optic disc vessel density measurement by using Spectral-domain OCT-A (Optovue Angiovue System, software ReVue XR version 2017.1.0.151).

Papilledema grading was done in accordance to the Modified Frise´n Scale [7].

### Optic disc vessel density measurement

Spectral-domain OCT-A (Optovue Angiovue System, software ReVue XR version 2017.1.0.151, Optovue) was used to measure optic disc vessel density. Measurements were done by the same one between 8-11 am to avoid the effect of diurnal variation.

A split-spectrum amplitude decorrelation algorithm (SSADA) is used to avoid motion artifacts and improve signal-to-noise ratio.

An ellipse was fitted automatically to the optic disc margin by the device software to measure the vessel density of the optic disc (known as inside disc vessel density). The peripapillary area was an area of 750 um that extends from the boundary of the optic nerve. The whole image vessel density was calculated in an area of 4.5×4.5mm around the disc as well as the vessel density in the peripapillary area.

Bad-quality images with signal strength less than 70% were excluded. Also, images with motion or segmental artifact were excluded.

### Re-evaluation

All patients underwent re-evaluation after 1 week, 1month and 3 months postoperatively. Fundus examination, neurological assessment (clinical follow up of symptoms headache, blurring of vision, fundus examination), and measuring the optic disc vessel density were performed.

Then the patients were divided in accordance to fundus examination into 2 groups: 1) the completely resolved disc oedema group, and 2) the residual disc elevation group.

Special investigation for patients with residual papilledema; Plain X-ray lumbosacral spine AP and lateral to ensure the site of the shunt in case of lumboperitoneal shunt.

Lumbar puncture was done for all cases of residual disc elevation to measure CSF opening pressure. Patients with normal opening pressure with residual disc elevation were compared with patients with completely resolved disc oedema.

Pelvi abdominal CT mylogram for cases of lumboperitoneal shunts to ensure shunt function was done.

### Statistical analysis

The Statistical Package for Social Science (IBM SPSS) version 23 was used to analyze data after revising and codding. The data was represented in the form of mean, standard deviation and the range. Chi-square test and Independent t-test were used to compare between the two groups. The confidence interval was set to 95% and the margin of error accepted was set to 5%. So, the p-value was considered significant at level of < 0.05.

## Results

Fifteen patients (71.4%) had a completely resolved disc oedema, while 6 patents (28.6%) had a residual disc elevation.

There was a non-statistically significant difference between the two groups as regard age (with a P-value 0.502). There was a highly statistically significant difference between the two groups as regard the papilledema grade (the residual disc elevation group had a higher grade of preoperative papilledema) with a P- value 0.000 (Table 1).

**Table 1:** Comparison between the residual disc elevation group and the completely resolved disc oedema group

		The residual disc elevation group	Completely resolved disc oedema group	Test value	P-value	Sig.
		No. = 6	No. = 15			
Age	Mean ± SD	36.00 ± 4.18	34.40 ± 7.71	0.677•	0.502	NS
	Range	32 – 40	27 – 48			
Grade of papilledema	Grade II	0 (0.0%)	15 (50.0%)	21.000*	0.000	HS
	Grade III	6 (50.0%)	15 (50.0%)			
	Grade IV	6 (50.0%)	0 (0.0%)			
VA	Mean ± SD	0.38 ± 0.14	0.74 ± 0.10	-9.422•	0.000	HS
	Range	0.2 – 0.5	0.6 – 0.9			
MD	Mean ± SD	-11.43 ± 11.27	-9.10 ± 4.90	-0.944•	0.351	NS
	Range	-22.9 – 0	-21.3 – -5.4			
LP	Mean ± SD	37.50 ± 2.74	34.60 ± 4.22	1.544•	0.139	NS
	Range	35 – 40	30 – 40			
MRV	Normal	3 (50.0%)	6 (40.0%)	3.850*	0.146	NS
	TS hypoplasia	3 (50.0%)	3 (20.0%)			
	SSS thrombosis	0 (0.0%)	6 (40.0%)			
Complication	No	6 (100.0%)	6 (40.0%)	6.300*	0.012	S
	Intracranial hypotension	0 (0.0%)	3 (20.0%)	1.400*	0.237	NS
	Wound infection	0 (0.0%)	3 (20.0%)	1.400*	0.237	NS
	Intestinal injury	0 (0.0%)	3 (20.0%)	1.400*	0.237	NS

*: Chi-square test; •: Independent t-test

There was a no statistically significant difference between the two groups as regard preoperative values of CSF opening pressures between the two groups.

As regard the visual acuity, there was a highly statistically significant difference between the two groups with worse preoperative visual acuity in the residual disc elevation group with a P-value 0.000. However, there was a non-statistically significant difference between the two groups as regard the main deviation (MD) in the visual field with a P-value 0.351 (Table 1). The types of visual field defect were enlargement of blind spot (18 cases 85.7%) and generalized depression (4 cases 14.2%). Color vision was not affected in all cases.

There was no statistically difference between the two groups as regard the pre-operative total retinal nerve fiber layer (RNFL) with a p-value 0.327 (Table 2). As regard the post-operative total RNFL, there was a highly statistically significant difference between the both group with P- value 0.0017 (Table 2).

**Table 2:** The compression of the retinal fiber nerve layer ( RNFL) between the residual disc elevation group and the completely resolved disc oedema group

		The residual disc elevation group	Completely resolved disc oedema	Test value	P-value	Sig.
		No. = 12 eyes	No. = 30eyes			
Pre-operative total RNFL	Mean ± SD	138.83±10.19	133.17±8.86	1.026	0.328	NS
Post-operative total RNFL	Mean ± SD	126.50±3.87	113.00± 3.16	5.400	0.0017	HS

**Fig 1:**
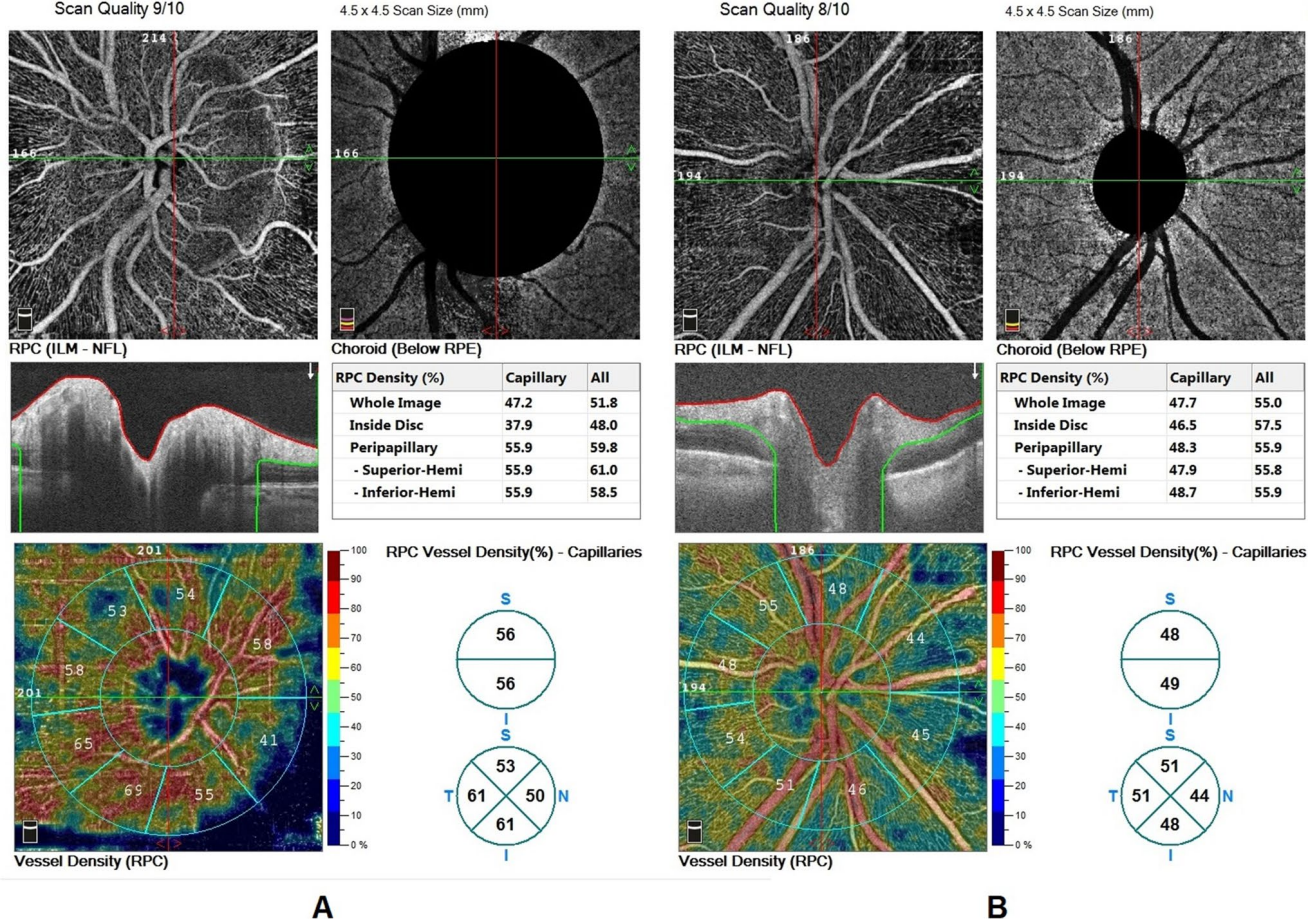
**A**) optic disc vessels density of papilledema before surgery, **B**) optic disc vessel density after surgery with residual disc oedema

In the residual disc elevation group, there were highly statistical changes in optic disc vessel density after shunt surgery in microvasculature (referred to as capillary vessel density) and macrovasculatrure (referred to as all vessel density). These changes occurred in whole image, inside disc, peripapillary, superior-hemisphere and inferior-hemisphere vessel density (Table 3) (Fig. 1).

**Table 3:** The per and post-operative optic disc vessel density in the residual disc elevation group

The residual disc elevation group with Normal LP cases		Pre	Post	Mean diff. ± SD	Test value	P-value	Sig.
Whole image(cap)	Mean ± SD	53.95 ± 2.49	47.73 ± 2.90	-6.23 ± 1.36	15.803•	0.000	HS
	Range	49.9 – 55.9	43.4 – 50.4				
Whole image (all)	Mean ± SD	59.88 ± 0.68	54.03 ± 2.54	-5.85 ± 2.01	10.070•	0.000	HS
	Range	58.9 – 60.6	51.2 – 57.2				
Inside disc (cap)	Mean ± SD	59.28 ± 6.40	46.05 ± 3.70	-13.23 ± 8.70	5.267•	0.000	HS
	Range	51.2 – 68.4	41.3 – 51.3				
Inside disc (all)	Mean ± SD	67.58 ± 6.65	56.60 ± 4.20	-10.75 ± 8.03	4.508•	0.001	HS
	Range	59.4 – 76.7	51.8 – 62.5				
Peripapillary (cap)	Mean ± SD	58.98 ± 1.16	53.35 ± 1.44	-5.63 ± 0.98	19.923•	0.000	HS
	Range	57.2 – 60.1	51.5 – 55				
Peripappillary (all)	Mean ± SD	62.05 ± 2.57	55.48 ± 6.48	-6.58 ± 4.20	5.420•	0.000	HS
	Range	58.4 – 64.4	44.9 – 60.3				
Superior- hemi (cap)	Mean ± SD	57.43 ± 2.80	52.05 ± 2.31	-5.38 ± 0.78	23.782•	0.000	HS
	Range	52.8 – 59.3	48.4 – 54				
Superior- hemi (all)	Mean ± SD	61.83 ± 3.43	57.38 ± 3.01	-4.45 ± 1.02	15.044•	0.000	HS
	Range	57.7 – 65.7	54.3 – 60.3				
Inferior-hemi (cap)	Mean ± SD	58.20 ± 2.15	51.83 ± 2.15	-6.38 ± 0.21	103.324•	0.000	HS
	Range	54.8 – 59.9	48.4 – 53.7				
Inferior- hemi (all)	Mean ± SD	62.80 ± 2.74	55.33 ± 6.61	-7.48 ± 4.31	6.008•	0.000	HS
	Range	59.3 – 65.8	44.7 – 60.2				

•: Independent t-test

There was a statistically significant difference between the two groups as regard the difference in the preoperative optic disc vessel density (optic disc vessel density was more in the residual disc elevation group) (Table 4).

**Table 4:** The compression of the pre-operative optic disc vessel density between the residual disc elevation group and the completely resolved disc oedema group

Pre		The residual disc elevation group	Completely resolved disc oedema	Test value	P-value	Sig.
		No. = 12eyes	No. = 30eyes			
Whole image(cap)1	Mean ± SD	54.85 ± 2.29	47.83 ± 16.55	-2.308•	0.026	S
	Range	50.9 – 58.5	42.7 – 55.9			
Whole image (all) 1	Mean ± SD	58.20 ± 2.15	55.21 ± 2.53	3.595•	0.001	HS
	Range	54.8 – 59.9	50.3 – 59			
Inside disc (cap) 1	Mean ± SD	59.28 ± 6.40	54.93 ± 3.25	2.925•	0.006	HS
	Range	51.2 – 68.4	49.7 – 60.3			
Inside disc (all)1	Mean ± SD	67.58 ± 6.65	63.94 ± 3.21	2.401•	0.021	S
	Range	59.4 – 76.7	59.1 – 68.9			
Peripapillary (cap)1	Mean ± SD	58.98 ± 1.16	55.07 ± 2.76	4.710•	0.000	HS
	Range	57.2 – 60.1	50.2 – 59.7			
Peripappillary (all)1	Mean ± SD	62.80 ± 2.74	61.16 ± 1.46	2.527•	0.016	S
	Range	59.3 – 65.8	58.4 – 63.7			

•: Independent t-test

There were non-significant differences between the two groups in postoperative optic disc vessel density in whole image, inside disc and peripapillary vessel density (either in macro or microvasculature) (Table 5).

**Table 5:** The compression of the post-operative optic disc vessel density between the residual disc elevation group and the completely resolved disc oedema group

POST		The residual disc elevation group	Completely resolved disc oedema	Test value	P-value	Sig.
		No. = 12eyes	No. = 30eyes			
Whole image (capillary)	Mean ± SD	47.08 ± 3.13	48.40 ± 2.07	-1.611•	0.115	NS
	Range	43.4 – 51.3	44.4 – 51.4			
Whole image (all)	Mean ± SD	54.03 ± 2.54	54.78 ± 2.13	-0.982•	0.332	NS
	Range	51.2 – 57.2	50.4 – 57.1			
Inside disc (capillary)	Mean ± SD	46.05 ± 3.70	47.71 ± 3.42	-1.387•	0.173	NS
	Range	41.3 – 51.3	43.1 – 53.2			
Inside disc (all)	Mean ± SD	56.60 ± 4.20	57.92 ± 2.99	-1.148•	0.258	NS
	Range	51.8 – 62.5	53.1 – 61.2			
Peripapillary (capillary)	Mean ± SD	55.33 ± 6.61	55.77 ± 2.70	-0.313•	0.756	NS
	Range	44.7 – 60.2	51.3 – 60.2			
Peripappillary (all)	Mean ± SD	55.48 ± 6.48	54.93 ± 2.54	0.396•	0.694	NS
	Range	44.9 – 60.3	51.2 – 59			

•: Independent t-test

As regard the reduction in optic disc vessel density, there were statistically significant differences between both groups with more reduction in optic disc vessel density in the residual disc elevation group (Table 6).

**Table 6:** The compression of the difference in optic disc vessel density between the residual disc elevation group and the completely resolved disc oedema group

difference		The residual disc elevation group	Completely resolved disc oedema	Test value	P-value	Sig.
		No. = 8 eyes	No. = 30eyes			
Whole image(cap)	Mean ± SD	-7.80 ± 2.71	-5.62 ± 1.09	-3.752•	0.001	HS
	Range	-11.9 – -4.3	-8.2 – -4.2			
Whole image (all)	Mean ± SD	-6.38 ± 1.66	-4.71 ± 1.16	-3.705•	0.001	HS
	Range	-8.5 – -3.4	-6.6 – -3.2			
Inside disc (cap)	Mean ± SD	-13.23 ± 8.70	-7.22 ± 3.36	-3.266	0.002	HS
	Range	-27.1 – -5.6	-13.9 – -3.6			
Inside disc (all)	Mean ± SD	-10.75 ± 8.03	-6.02 ± 1.79	-3.093	0.004	HS
	Range	-24 – -5.1	-9.4 – -3.3			
Peripapillary (cap)	Mean ± SD	-6.08 ± 1.29	-4.24 ± 1.64	-3.451•	0.001	HS
	Range	-8.7 – -4.6	-6.8 – -1.1			
Peripappillary (all)	Mean ± SD	-7.75 ± 3.81	-5.82 ± 1.14	-2.545•	0.015	S
	Range	-13.5 – -3.9	-8.1 – -3.9			

•: Independent t-test

As regards cases of residual papillidema, lumber puncture was normal and correlated with improved optic disc vessel density. Furthermore, pelvi-abdominal CT mylogram was normal which ensured good functioning shunt.

We have one case with a longer postoperative follow up period for about 9 months the patient firstly after 3months improved clinically as regards headache ,blurring of vision ,papilledema improved from grade 3 preoperative to grade 1 postoperative after 3moths follow up correlated with postoperative decreased optic disc vessel density. Lumber puncture was done and CSF opening pressure was normal.

Six months later (9 months postoperative) the patient starts to develop recurrent attacks of severe headache, blurring of vision fundus examination done revealed higher degree of papilledema 3rd degree, pelvi-abdominal CT revealed migrated shunt end (shunt malfunction). OCT-A revealed increase optic disc vessel density, CSF opening pressure was high about 40mmhg. All these data ensured that optic disc vessel density measurement can help fundus examination for follow up of postoperative cases of IIH, when there is confusion by residual papilledema. But we need further studies and more patients to ensure this result.

## Discussion

The pathogenesis of papilledema in IIH is mainly due to the mechanical effect. Vascular affection occurs secondary to these mechanical changes. The optic nerve and brain share the same leptomeninges. When CSF pressure increases, it compresses the optic nerve leading to stasis of axoplasmic flow of the optic nerve fiber layer and the optic disc. This stasis in the axoplasmic flow leads to swelling of nerve fiber, and subsequently the optic disc. This swelling secondarily compresses the optic disc venules leading to leakage of extracellular fluid. Thus, vascular changes in papilledema are secondary and not primary [9].

The visual impairment most likely is not due to axoplasmic stasis only because the impulses are conducted through the axon membrane and not through the axoplasm. Thus, vascular changes play a role in the visual impairment [7].

So, we can use the secondary vascular changes that occur in papilledema to evaluate the effect of intracranial pressure reduction after shunt surgery.

The sure method to detect intracranial pressure is by measuring the CSF opening pressure but it is an invasive method. So, many non-invasive methods were used to detect increasing intracranial pressure [10].

We can use papilledema resolution as an indicator of the reduction of CSF opening pressure. However, Sinclair et al [11] reported that papilledema was completely resolved only in 44% of patients, which means that about 56% of patients may have residual optic disc elevation.

After normalization of ICP by medical treatment or surgery, papilledema resolves within week or months. However, some patients have residual disc elevation which may occur as a result of gliosis in the optic disc [1]. The delay in the resolution of disc oedema may be due to congenital variation in the distance of the intracanlicular part of the optic nerve and the size of the lamena creprosa opening [7].

Fundus examination and fundus photography are good methods to detect papilledema resolution. However, in some situations, depending on them is difficult. In cases with optic disc morphological abnormality (eg. Tilted disc) it is difficult to distinguish between disc oedema and blurring of the disc margin. In longstanding papilledema, gliosis of the optic disc occurs making the optic disc margin irregular which subsequently leads to difficult papilledema recognition by fundus examination [12] In recurrent and relapsed IIH, detection of papilledema by fundus examination is difficult as a result of the gliosis and thinning of RNFL that occurred from the previous attack [8].

In those patients, we need to find a method to ensure that optic nerve is in a safe condition as a confirmatory method with measurement of CSF opening pressure by lumber puncture and in the future it may replace the invasive lumber puncture.

Depending on clinical follow up alone isn’t conclusive as in some patients they suffer postoperative headache of low intracranial tension but the patient usually fails to declare it obviously. In cases this headache is associated with residual papilledema it is crucial to investigate thoroughly to be sure that intracranial tension is normal. Normal CSF opening pressure by Lumber puncture alone indicates that intracranial tension is normal but it is invasive method and in case of residual papilledema .it is a must to ensure that optic nerve is in a safe condition.

MRI optic nerve is crucial in the diagnosis of IIH. However, the morphological signs of MRI become clearly visible only in advanced disease stages on conventional MRI. So, it can’t be used in early detection or follow-up of patients [13, 14]. Ultrasound is useful in differentiating papilledema from psudopapilledema. It can measure the morphological changes in optic nerve like MRI [15]. However, these changes have low specificity and need highly expert examiners [9]. Moreover, measuring the retinal nerve fiber layer thickness by OCT can be used to detect papilledema. However, depending on it in papilledema follow-up is somewhat problematic. Decreasing the RNFL thickness may be due to a true reduction of the optic disc oedema. Yet, it may be due to atrophy of RNFL that may occur during the course of the disease [7].

In this study, we performed shunt surgery for patients with IIH. Then we divided the patients after surgery into a completely resolved papilledema group and a residual disc elevation group. Both groups have successful surgery proved clinically by resolution of preoperative symptoms of headache, blurring of vision and vomiting, fundus examination which revealed resolution of papilledema in 15 cases and residual papilledema in 6 patients, those 6 patients were submitted to measuring the post-operative CSF opening pressure by lumber puncture which was normal for all patients.

In the residual disc elevation group, the optic disc vessel density was significantly reduced after surgery. The normal CSF opening pressure indicates resolution of IIH. So, the reduction in optic disc vessel density can be used as an indicator for the resolution of IIH in cases with residual disc elevation.

In this study, the grade of papilledema in the residual disc elevation group is higher than the completely resolved papilledema. Thus, it may take a longer duration to resolve.

The absence of significant differences between the two groups as regard the post-operative optic disc vessel density; indicates that the vessel density can help in cases of residual disc elevation.

To our knowledge, this is the first study that detects the reduction of optic disc vessel density after shunt surgery for patients with IIH having a residual elevation of the optic disc.

There are some limitations of measuring the optic disc vessel density by OCT-A. In severe degrees of papilledema and a high degree of myopia; the quality of the image is poor; making the quantitative measure of vessel density difficult. Also, media opacity can hinder light from reaching the retina, (fundus examination can’t be done in these cases also). Moreover, pregnancy, smoking, and some medications can affect vessel density measurement. However, we can depend on the difference between the pre and postoperative values.

In conclusion, in cases of residual optic disc swelling after shunt surgery, we may detect the reduction of intracranial pressure by the reduction in the optic disc vessel density which is a safe non-invasive technique. That may replace the invasive CSF opening pressure measurement. Still we need more patients and more studies to prove this evidence.
